# Lactulose Hydrogen Breath Test Result Is Associated with Age and Gender

**DOI:** 10.1155/2016/1064029

**Published:** 2016-03-17

**Authors:** Carolyn Newberry, Ann Tierney, Octavia Pickett-Blakely

**Affiliations:** ^1^University of Pennsylvania Perelman School of Medicine, Division of Gastroenterology, Philadelphia, PA 19104, USA; ^2^University of Pennsylvania Center for Clinical Epidemiology and Biostatistics, Philadelphia, PA 19104, USA

## Abstract

Small intestinal bacterial overgrowth (SIBO) is associated with chronic gastrointestinal diseases and structural/functional abnormalities of the gastrointestinal tract. SIBO's association with clinical characteristics is unclear. This study investigates the association between clinical factors and SIBO according to lactulose hydrogen breath test (LHBT) result.* Methods*. A cross-sectional study in a university-based gastroenterology practice was performed. Data was abstracted from the medical records of subjects undergoing LHBT from 6/1/2009 to 6/1/2013. Logistic regression analysis was performed to determine the association between predictor variables: age, sex, body mass index (BMI), and positive LHBT, the outcome of interest.* Results*. LHBT was performed in 791 subjects. Fifty-four percent had a positive LHBT. There was no statistically significant difference between the LHBT results according to age or BMI. In females, the likelihood of a positive LHBT increased with age (OR 1.02; 95% CI: 1.01–1.03). In males, the likelihood of a positive LHBT result decreased with age (OR 0.98; 95% CI: 0.97–1.00).* Conclusion*. There was an association between age, with respect to sex, and a positive LHBT. With increased age in females, the odds of a positive LHBT increased, while, in men, the odds of a positive LHBT decreased with age.

## 1. Introduction

Although the pathophysiology is poorly understood, small intestinal bacterial overgrowth (SIBO) is not uncommon with reported prevalence ranging from 37.5 to 50% in selected patient populations [1–3]. Clinical manifestations of the disease vary widely, from mild gastrointestinal symptoms such as flatulence and bloating to more serious complications including profound weight loss and micronutrient deficiencies [4]. Development of the condition may result after disruption of the homeostatic mechanisms that manage enteric bacterial colonies including gastric acid secretion, intestinal motility, anatomical structures such as the ileocecal valve, and immunomodulation [5]. For this reason, several gastrointestinal conditions have been associated with SIBO including inflammatory bowel disease (IBD) [6, 7], irritable bowel syndrome (IBS) [1, 8], motility disorders such as gastroparesis [3], and celiac disease [9]. Previous gastrointestinal surgery [10] and demographic factors including increasing age have also been implicated [11, 12].

In light of the evolving spectrum of SIBO-related clinical associations and manifestations as well as the availability of noninvasive diagnostic testing, health care providers are now testing patients for SIBO in increasing numbers [5]. Although the gold standard for diagnosis of the disease is a small bowel aspirate and culture, this method is invasive and challenging due to the difficultly associated with culturing gut flora [13, 14]. As a result of these limitations, noninvasive testing has been developed utilizing indirect means of assessing the presence of excessive numbers of bacterial colonies in the small bowel. The hydrogen breath test consists of administration of soluble and insoluble agents such as glucose and lactulose, the latter of which is preferentially metabolized by gut flora, with subsequent measurement of expired hydrogen and methane gas production [13]. Diagnostic criteria are not standardized but include a specified rise in hydrogen or methane levels within a given time period, usually 90–120 minutes after glucose or lactulose administration, which reflects carbohydrate fermentation within the small bowel [3, 15].

Currently, there is limited data examining the association between SIBO and demographic factors such as age and sex and clinical factors such as obesity. It is therefore the aim of this study to investigate the association between age, sex, body mass index (BMI), and lactulose hydrogen breath test, a surrogate marker for SIBO.

## 2. Methods

### 2.1. Patient Population and Data Collection

Consecutive subjects undergoing hydrogen lactulose breath test (LHBT) between 6/1/2009 and 6/1/2013 in the Gastroenterology Division at the University of Pennsylvania were eligible for study inclusion. Data collection was completed utilizing the electronic medical record to abstract demographic and clinical information. This study was approved by the Institutional Review Board at the University of Pennsylvania, Philadelphia, Pennsylvania.

### 2.2. Hydrogen Lactulose Breath Test

All subjects who underwent LHBT (indications ranged from bloating to diarrhea) at the University of Pennsylvania were instructed to refrain from antibiotic use for 14 days, to discontinue probiotics for 7–10 days, and not to take any home medications for 2 hours prior to testing. In addition, dietary avoidance of fruits, vegetables, grains, beans, bran cereals, and smoking for at least 24 hours prior to the procedure was recommended with complete fasting required 12 hours before testing. Sleeping and smoking were also prohibited within 30 minutes of testing. During the test, lactulose was administered to patients with subsequent breath samples acquired in 15-minute intervals for the duration of 180 minutes. Breath testing was performed using Quintron gas chromatography. Based on previously published literature, a positive LHBT was defined by one of the following criteria: (1) >10 ppm rise in methane or ≥20 ppm rise in hydrogen within the first 90 minutes of lactulose administration or (2) baseline H2 ≥ 20 ppm or CH4 ≥ 10 ppm [13, 16].

### 2.3. Analysis

Descriptive statistics were performed comparing characteristics of the study sample with a positive and negative LHBT. Univariable and multivariable logistic regression analysis were performed to examine the association of the predictor variables and the outcome of interest. The primary outcome variable was LHBT result (positive or negative). The predictor variables were age, sex, and BMI. Age and weight status (BMI) were treated as continuous and dichotomous variables. Age was categorized as greater than or equal to 65, established as definition of older age, and less than 65. BMI was calculated by the standard formula as weight/height in kg/m^2^. Patients were categorized as obese if BMI was ≥30, as defined by the WHO classification system [17]. Models were adjusted for proton pump inhibitor use and prior gastrointestinal luminal surgery (including partial or complete gastrectomy, small bowel resection, partial or complete colonic resection, gastric bypass, and pancreaticoduodenostomy) based on preliminary chi-square analysis. Statistical calculations were conducted using SAS 9.4. A *P* value of <0.05 was used to define statistical significance in the final models.

## 3. Results

### 3.1. Study Sample Characteristics

A total of 791 adult subjects were included in the study.  Table 1 shows the characteristics of the study sample. Most patients were female (69.8%), under the age of 65 (79.8%), and not obese (62.5%). The mean age at the time of LHBT was 49 years (SD 17.1). The mean BMI was 26.9 kg/m^2^ (SD 6.1). In addition, 56% of subjects reported recent proton pump inhibitor use (listed as an active medication at the time of the LHBT), 4.6% were diagnosed with gastroparesis, 8.3% were diagnosed with IBD, 1.5% were diagnosed with celiac disease, and 16.2% had prior gastrointestinal tract surgeries.

### 3.2. Demographic and Clinical Associations with SIBO

Based on the aforementioned diagnostic criteria, 54.4% of subjects had a positive LHBT.  Table 2 displays the results of the association between age, sex, BMI, and LHBT result. With respect to age, there was no significant association between LHBT results in those over the age of 65 compared with those under the age of 65 (OR 1.38, 95% CI 0.97–1.96). Age examined continuously was also not associated with a positive LHBT (OR 1.005, 95% CI 1.00–1.01). When examining sex alone, there was also no association between LHBT results (OR 1.18, 95% CI 0.87–1.60). However, in females, as age increased, there were significantly increased odds of having a positive LHBT compared with a negative LHBT (OR 1.02, 95% CI 1.01–1.03). In males, as age increased, there were decreased odds of having a positive LHBT compared with a negative LHBT (OR 0.98, 95% CI: 0.97–1.00). BMI category (obese versus nonobese) was not associated with LHBT result (OR 0.85, 95% CI: 0.62–1.16). However, when explored in a continuous fashion, every 1 unit increase in BMI was associated with a 3% decrease in the likelihood of having a positive LHBT (OR 0.97, 95% CI 0.95–0.99), suggesting an inverse association.

In multivariable models adjusting for other important predictors associated with a positive LHBT in this cohort, including proton pump inhibitor use and a history of gastrointestinal surgery, there remained a significant interaction with age and sex (OR 1.04, 95% CI 1.02–1.05). For women, as age increased, there was an increase in the odds of having a positive LHBT (OR 1.02, 95% CI 1.01–1.03). BMI examined in a continuous fashion remained a significant negative predictor of a positive LHBT and hence it was retained in the final model (OR 0.97, 95% CI 0.95–1.00).

## 4. Discussion

Our study showed that a positive LHBT was common in our cohort, suggesting that just over one-half of the study sample had SIBO as diagnosed by this modality. Furthermore, we found a significant association between sex with respect to age and LHBT result, with females being more likely to have a positive LHBT result as age increased and males being less likely to have a positive result as age increased, though there was no association between age alone and LHBT result. There was a small but significant decrease in the likelihood of having a positive LHBT as BMI increased.

The prevalence of a positive LHBT in our sample of patients being evaluated for gastrointestinal symptoms is comparable to previously reported estimates in other populations. For example, others have reported a positive LHBT in 50% of a cohort with recent PPI use [2], 37.5% of those with IBS [1], and 39% of those with gastroparesis [3]. Our analysis revealed an inverse association between prior gastrointestinal surgery and positive LHBT which is not consistent with the published literature [1, 2, 9, 11]. Of note, only one-third of those with prior gastrointestinal surgery involved the potential loss of the ileocecal valve. Retention of the ileocecal valve, an anatomic barrier to colonic flora entering the small bowel, may explain the inverse association observed in this study. Furthermore, we did not find an association between IBD, gastroparesis or celiac disease, and LHBT in unadjusted analyses, which differs distinctly from recently published studies noting such a relationship in IBD patients, particularly Crohn's disease [6, 7], proximal motility disorders like gastroparesis [3], and celiac disease [9]. It is possible that such a relationship was underestimated in our study group given that only 4.7% of our sample had gastroparesis, 8.3% had IBD, and 1.5% had celiac disease.

We also did not observe an association between elderly age (less than 65 versus greater than 65) and positive LHBT. This is not consistent with prior studies in the elderly that suggest an association between age and positive HBT [11, 12]. The lack of association between LHBT result and age in our study is surprising, given that intrinsic changes in intestinal motility, hypochlorhydria, development of small bowel diverticula, polypharmacy (which can affect gastrointestinal motility), and higher prevalence of comorbid conditions often afflict those over the age of 65 [4, 5, 14]. Furthermore, older age has been suggested as a risk factor of bacterial overgrowth in prior studies [7, 12]. Additionally, Schatz et al. showed a relationship between gender, age, and LHBT result in females. Females with a positive breath test were greater than 5 years older than those with a negative LHBT test [18]. The present analysis also did not find an association between obesity (BMI ≥ 30 kg/m^2^) and positive LHBT. There are published reports that contradict this finding including a study published by Sabaté et al. which showed an association between positive LHBT and morbid obesity in a population referred for bariatric surgery [10]. Basseri et al. also found elevation in methane levels by LHBT in their prospective trial of bariatric surgical candidates compared with normal weight controls [19]. It is possible that the severity of obesity plays a role in breath test result, as both of these studies examined patient populations with severe obesity (BMI ≥ 40 kg/m^2^) compared to our study sample in which we compared obese (BMI ≥ 30 kg/m^2^) and nonobese (BMI < 30 kg/m^2^) subjects. Of note, only 20 subjects in our study sample had a BMI of ≥40 kg/m^2^ which may have led to an underestimation of the odds of a positive LHBT at higher levels of BMI. However, when examined as a continuous variable, BMI was inversely associated with a positive breath test result. Interestingly, Parlesak and colleagues reported that BMI was inversely related to positive glucose breath test, particularly in the elderly. In this study, patients >60 years of age with positive LHBT had lower BMI, body weight, and nutritional markers including albumin and vitamin B-12 [12]. It is possible that clinical implications of SIBO in the older adult are more severe and lower body weight is a result of the condition, not a predisposition. This may be especially true in the elderly who often have a significant delay between symptom onset and clinical diagnosis in the setting of multiple comorbidities [11].

Sex was associated with LHBT result, but only when age was also taken into account. Our cohort was approximately 70% female, consistent with other studies examining SIBO prevalence in the general population [1, 7]. It is likely that higher rates of testing are reported in females as compared to males in the setting of increased rates of nonspecific gastrointestinal symptoms and functional bowel disorders [20]. Our study, in addition to that reported by Choung et al. [7], confirms that the higher screening rate does not necessarily indicate higher prevalence in females. We did, however, find that increasing age affects LHBT result differently in females versus males. This may be related to hormonal fluctuations, which have been shown in animal models to affect motility and gastric acid secretion [21–23].

Although our findings are interesting, this study does have several limitations. First, the definition of SIBO according to LHBT is not in accordance with clinical guidelines that implicate duodenal aspirate as the gold standard for diagnosis of SIBO. However, in the setting of cost considerations and the desire to test by noninvasive means, breath testing has surfaced as an alternative to endoscopic sampling. When choosing a substrate for breath tests, both glucose and lactulose are clinically available. Glucose provides the advantage of being rapidly metabolized in the proximal small intestine, thereby increasing sensitivity and specificity of the test in comparison to lactulose, which passes into the colon [13]. Glucose, however, does not detect methane-producing bacteria, which remains a small but important subset of bacterial colonization in SIBO patients. Our center uses lactulose LHBT for the diagnosis of SIBO based on these principles. We also recognize that the diagnostic criteria for LHBT are not standardized. Our positive test was defined by hydrogen peak > 20 ppm or methane > 10 ppm within a 90-minute period or a baseline hydrogen ≥ 20 ppm or methane ≥ 10 ppm [16], consistent with prior studies utilizing LHBT [3, 15]. We did not require a double peak as this criterion has not been validated as reported by Pimentel et al. [16]. In addition to testing modality, our study was a retrospective chart review, which is associated with selection and information bias. Given the nature of the tertiary care practice, it is also possible that the results herein are not generalizable to the general population and should be reproduced in other more inclusive populations.

This cross-sectional analysis is the first to examine the association between demographic and clinical factors and positive LHBT for the diagnosis of SIBO. This information, if confirmed, may aid practitioners in targeting diagnostic testing to higher risk populations such as aging females. Further investigation into the clinical and demographic associations that predispose patients to the development of SIBO (as detected by a positive LHBT) is necessary to guide providers in diagnosis of this disease.

*Additional Points*. The study highlights the following:

1. What is current knowledge?
  - Small intestinal bacterial overgrowth (SIBO) more commonly affects individuals with predisposing gastroenterological conditions.
  - SIBO causes a range of symptoms including diarrhea, bloating, and abdominal pain.
2. What is new here?
  - There are increased odds of having a positive lactulose breath test in women as age increases.
  - There are decreased odds of having a positive lactulose breath test in men as age increases.

## Figures and Tables

**Table 1:** Demographic and clinical characteristics of study population.

Characteristics	Positive HBT (*n* = 430)	Negative HBT (*n* = 361)	Overall (*n* = 791)	*P* value
Sex				0.28
Male (%)	123 (28.6)	116 (32.1)	239 (30.2)	
Female (%)	307 (71.4)	245 (67.9)	552 (69.8)	
Age (years, mean ± SD)	49.7 ± 17.3	48.2 ± 16.8	49.0 ± 17.1	0.07
<65 years (%)	333 (77.4)	298 (82.5)	631 (79.8)	
≥65 years (%)	97 (22.6)	63 (17.5)	160 (20.2)	
BMI (kg/m^2^, mean ± SD)	26.4 ± 5.7	27.4 ± 6.48	26.9 ± 6.1	0.30
<30 (%)	318 (74.0)	255 (70.6)	573 (72.4)	
≥30 (%)	112 (26.0)	106 (29.4)	218 (27.6)	
Disease presence				
Gastroparesis	15 (3.5)	22 (6.1)	37 (4.7)	0.08
IBD	30 (7.0)	36 (10.0)	66 (8.3)	0.13
Celiac disease	6 (1.4)	6 (1.7)	12 (1.5)	0.76
Other factors				
GI surgery history	59 (13.8)	69 (19.1)	128 (16.2)	**0.04**
Recent PPI use	213 (49.5)	208 (57.6)	421 (53.2)	**0.02**

**Table 2:** Clinical features associated with positive LHBT.

	Odds ratio	95% CI interval	*P* value
Univariable analyses			
Sex, F versus M	1.18	0.87–1.60	0.28
*Age *			
<65 versus ≥65 years	1.38	0.97–1.96	0.76
Continuous variable	1.01	1.00–1.01	0.23
*BMI *			
<30 versus ≥30 kg/m^2^	0.85	0.62–1.15	0.30
Continuous variable	0.97	0.95–1.00	0.03

Multivariable analyses			
Sex, F versus M	1.17	0.86–1.6	0.32
*Age *			
<65 versus ≥65 years	0.71	0.49–1.01	0.06
Continuous variable	1.005	1.00–1.01	0.11
*BMI *			
<30 versus >30 kg/m^2^	0.85	0.62–1.17	0.32
Continuous variable	0.97	0.95–1.00	0.15

Female/age	1.02	1.01–1.03	<0.001
Male/age	0.98	0.97–1.00	0.03
